# Progress in Surgery

**Published:** 1899-08-19

**Authors:** 


### Progress in Surgery.

### TUMOURS.

(*Continued from page* 332.)

**Cases of Special Interest.**—W. H. Bennett[9] has published a series of cases illustrating peculiarities in the behaviour of tumours. Some illustrate the occurrence of tumours apparently malignant which ran an innocent course; others are examples of tumours apparently innocent proving in the end to be malignant; and a third group illustrates the effect of concentration of the mind in the production of tumours innocent or malignant. A few of these may be briefly described. Case 1: A woman of 36 had in seven months become weak, greatly emaciated, and sallow, and had developed a tumour in the abdomen under the liver. The growth on opening the abdomen was found to be a nodulated mass of stony hardness, and appeared to invade the liver. On the upper surface of the latter was a separate nodule the size of half a walnut. The omentum was involved. A small piece was examined microscopically, and pronounced to be mixed round a spindle-celled sarcoma. Eighteen months later she was a well-nourished woman, and there was no trace of tumour. Case 2: A woman of 52 had a well-marked melanotic sarcoma of the sole of the foot excised. Later in the same year Syme's amputation was performed for recurrence. A month later there were some blackish tumours in the skin on the inner side of the thigh. These multiplied until 62 could be counted. There were at the same time hard glands in Scarpa's triangle. The primary and secondary growths were proved to be melanotic sarcomata microscopically. The case was regarded as hopeless. Subsequently all these nodules and the glands disappeared. Case 3 was similar to the last, except that the growth was on the dorsum of the foot. The nodules on the leg and the glands were in the popliteal region. Case 6 was that of a man in the last stage of emaciation, and of cachectic appearance, who had pyloric obstruction from a large growth. Gastro-enterostomy was performed. The mass in the pylorus was the size of a fist, and there were hard nodules in the omentum and under the liver. It was considered so clearly malignant that a piece was not removed for microscopical examination. The man was alive and in fair health five years after the operation, and had no perceptible tumour. Case 8: Castration for a testicular tumour, which the microscope showed to be encephaloid cancer, was performed. A hard mass formed a week later below the liver, and later one formed in the right iliac fossa. Six months later there was painless œdema of the right leg from pressure, and the case was considered hopeless. Six months later still, however, the iliac tumour had disappeared, and the other became very small. Case 9: In 1893 the left mamma of a woman was removed for scirrhus cancer. In 1896 the other breast was removed for the same disease. Soon after this some hard nodules appeared in the first scar, and ultimately about a dozen of these formed. Recently an examination showed that all the nodules had disappeared spontaneously. Cases 10 and 11 are of tumours of the mamma in females, clinically regarded as scirrhus, which did not actually disappear but became absolutely quiescent for many years, the patients dying of other ailments. Case 16 was that of a woman of 36, who for ten years had had a small fibro-adenoma of the breast. She had long ceased to pay any attention to this until a friend alarmed her by saying that cancer might result. From that time she thought much about it. Coincidently the tumour began to increase, and a month or two later was removed, being twice its original size. In case 17 a woman who greatly feared cancer had an adenoma removed, but she by day and night dwelt upon the possibility of the scar becoming cancerous, and six months later a cancer actually developed near the scar and adherent to it. It appears from the above cases that in a certain percentage of new growths the microscopical characters are not a reliable index to malignancy or the reverse. Albert (*Wien. Med. Woch.*, January, 1899)[10] reports four cases of cancer of both breasts, which he has met with among many hundreds of cases of that disease. Hansy (*ibid* Feb.) reports a case of the double disease in a male. The patient was a postman of 61, with a large ulcerated cancer of the left nipple, which had existed about a year, with corresponding disease on the right side; the latter had grown with great rapidity, and ulcerated. Both sides were operated upon, but the patient died of matastasis in eight months.

At the February meeting of the Pathological Society Stanley Boyd showed sections of a new growth of the rectum which had the clinical character of a columnar epithelioma, but histologically seemed to be an alveolar sarcoma. The patient was a woman of 35. Three days after colotomy the cæcum perforated, though the obstruction had been completely relieved. She died 30 days after this, chiefly from dyspnœa from accumulation of gas in the abdomen. This gas was contained in a huge cavity lined with lymph, the viscera were pressed behind this and were healthy. Dr. Hebb showed a specimen of colloid cancer of the stomach from a woman aged 36, who had had pain and vomiting for some months previous to death. The walls of the stomach were infiltrated with the growth, and the pyloric half ulcerated. Some neighbouring glands were carcinomatous, but with no colloid change.

[9] Lancet, Jan., 1899. [10] Brit. Med. Jour., Mar., 1899.

